# Real-time quantification of the transmission advantage associated with a single mutation in pathogen genomes: a case study on the D614G substitution of SARS-CoV-2

**DOI:** 10.1186/s12879-021-06729-w

**Published:** 2021-10-07

**Authors:** Shi Zhao, Jingzhi Lou, Lirong Cao, Hong Zheng, Marc K. C. Chong, Zigui Chen, Renee W. Y. Chan, Benny C. Y. Zee, Paul K. S. Chan, Maggie H. Wang

**Affiliations:** 1 JC School of Public Health and Primary Care, Chinese University of Hong Kong, Hong Kong, China; 2 CUHK Shenzhen Research Institute, Shenzhen, China; 3 Department of Microbiology, Chinese University of Hong Kong, Hong Kong, China; 4 Department of Paediatrics, Chinese University of Hong Kong, Hong Kong, China; 5 Hong Kong Hub of Pediatric Excellence, Chinese University of Hong Kong, Shatin, N.T., Hong Kong, China; 6 CUHK-UMCU Joint Research Laboratory of Respiratory Virus & Immunobiology, Chinese University of Hong Kong, Shatin, N.T., Hong Kong, China; 7 Li Ka Shing Institute of Health Sciences, Faculty of Medicine, Chinese University of Hong Kong, Shatin, N.T., Hong Kong, China

**Keywords:** COVID-19, Mutation, Transmission advantage, Real-time estimation, Statistical modelling

## Abstract

**Background:**

The COVID-19 pandemic poses serious threats to global health, and the emerging mutation in SARS-CoV-2 genomes, e.g., the D614G substitution, is one of the major challenges of disease control. Characterizing the role of the mutation activities is of importance to understand how the evolution of pathogen shapes the epidemiological outcomes at population scale.

**Methods:**

We developed a statistical framework to reconstruct variant-specific reproduction numbers and estimate transmission advantage associated with the mutation activities marked by single substitution empirically. Using likelihood-based approach, the model is exemplified with the COVID-19 surveillance data from January 1 to June 30, 2020 in California, USA. We explore the potential of this framework to generate early warning signals for detecting transmission advantage on a real-time basis.

**Results:**

The modelling framework in this study links together the mutation activity at molecular scale and COVID-19 transmissibility at population scale. We find a significant transmission advantage of COVID-19 associated with the D614G substitution, which increases the infectivity by 54% (95%CI: 36, 72). For the early alarming potentials, the analytical framework is demonstrated to detect this transmission advantage, before the mutation reaches dominance, on a real-time basis.

**Conclusions:**

We reported an evidence of transmission advantage associated with D614G substitution, and highlighted the real-time estimating potentials of modelling framework.

**Supplementary Information:**

The online version contains supplementary material available at 10.1186/s12879-021-06729-w.

## Introduction

The dynamics of the transmission of an infectious disease is largely determined by the pathogen’s infectiousness and the course of the transmission [1, 2]. The control of a contagious disease with high infectiousness requires the knowledge of the driven factors that may affect the transmission process [3, 4]. Virus mutation is one of the major challenges for controlling epidemics [5, 6]. The profile of pathogen in terms of viral fitness and functionality may be altered by mutations [7, 8], and in consequence change its transmissibility. Referring to the previous literature on seasonal influenza [9], a few key amino acid (AA) substitutions may lead to remarkable changing dynamics of antigenic property and epidemiological outcomes at population scale [10, 11]. Similar findings were also reported for other viral pathogens [12, 13].

The coronavirus disease 2019 (COVID-19), whose etiological agent is the severe acute respiratory syndrome coronavirus 2 (SARS-CoV-2) [14], swept the world in a short period of time [15], and the ongoing COVID-19 pandemic poses serious threat to public health [16]. As of December 31, 2020, over 81 million COVID-19 cases are confirmed in the world with over 1.8 million associated deaths. In February 2020, genetic variants carrying the D614G substitution on the SARS-CoV-2 spike (S) protein began to spread first in Europe [17] and otherwhere globally, reaching fixation in many places rapidly. The D614G is potentially affecting viral transmission [5, 18]. Recent modelling analysis reported statistical evidence that SARS-CoV-2 strains with D614G substitution are likely to have an increased infectivity retrospectively [19]. In 2021, although 614D still can be detected in some places, e.g., Australia with around 25% frequency, the variants carrying 614G is predominant globally.

Some of these variant genomes upon the different selection pressure increase their frequency in the population. Recently, the SARS-CoV-2 Delta variants composed of several novel mutations on Spike protein increased their frequency [20]. This becomes one of the major challenges of COVID-19 control because these variants have more competitive pathological features such higher transmission or resistance to vaccines [21, 22]. Exploring the relationship between the mutation activities and the disease transmissibility is of importance to understand how the evolutionary patterns at molecular scale may shape the epidemiological outcomes at population scale. Quantifying the advantage of mutations that affects the transmission may inform the disease control strategic decision-making process [23].

Given the intensity and the risk scale of the ongoing COVID-19 pandemic, real-time surveillance and inference of the role of key mutations may be crucial for fighting against the pandemic. In this study, we adopted a statistical inference framework to estimate the transmission advantage associated with a single mutation in pathogen genomes empirically, and exemplify by using the COVID-19 data in California, USA. We demonstrate the potentials of this analytical framework to produce an early warning signal for detecting transmission advantage on a real-time basis.

## Methods

### Reproduction number and transmission advantage: parameterization and likelihood framework

The time-varying reproduction number is commonly adopted to quantify the instantaneous transmissibility of infectious disease in an epidemic. Using the estimation framework in [24], the epidemic growth is modelled as a branching process, thus can be expressed as the ratio of *C*(*t*) over ${\int }_{0}^{\infty }w\left(k\right)C\left(t-k\right)\mathrm{d}k$, which is commonly known as the renewable equation [25]. Here, the *C*(*t*) is the observed number of new COVID-19 cases on the *t*-th day. The function *w*(∙) is the distribution of the generation time (GT) of the disease. The GT is defined as the time interval between the time of exposure, i.e., being infected, of a primary case and that of his associated secondary case in the consecutive transmission generation [26]. The distribution *w*(∙) is predefined in our model, which is commonly estimated from contract tracing surveillance data [27–30].

The transmission advantage of the mutated variant against the original type is defined as the ratio, denoted by *η*, of the strain-specified reproduction numbers. We denote the reproduction number of cases infected by the original variant as *R*_*t*_, and thus the reproduction number of cases infected by the mutated variant is *η*∙*R*_*t*_. If *η* > 1, the mutated variant may be more infectious than the original genetic variant, and vice versa.

The observed proportion of original genetic variant is denoted by *q*_*t*_, and the observed proportion of mutated variant is denoted by *p*_*t*_. Since we consider the binary AA substituting process, we have *p*_*t*_ + *q*_*t*_ = 1 for all *t*s. By using the renewable equation backwardly, we model the expected number of cases on the *t*-th day in Eq. (1).

1 $$\mathbf{E}\left[{C}_{t}\right]={R}_{t}\cdot \left[{\int }_{0}^{\infty }w\left(k\right)\cdot q\left(t-k\right)\cdot C\left(t-k\right)\mathrm{d}k+\eta {\int }_{0}^{\infty }w\left(k\right)\cdot p\left(t-k\right)\cdot C\left(t-k\right)\mathrm{d}k\right].$$

Here, the **E**[∙] denotes the expectation function. Therefore, we construct the likelihood function ${L}_{t}^{(\mathrm{c})}$ of the daily number of cases using a Poisson-distributed framework with observation at *C*_*t*_ and rate parameter at **E**[*C*_*t*_] as in Eq. (2).

2 $${L}_{t}^{(\mathrm{c})}\left({C}_{t}|\mathbf{E}\left[{C}_{t}\right]\right)=\frac{{\mathbf{E}\left[{C}_{t}\right]}^{{C}_{t}}\cdot {e}^{-\mathbf{E}\left[{C}_{t}\right]}}{{C}_{t}!}.$$

Here, the superscript ‘^(c)^’ merely indicated the likelihood function is for the number of cases, which does not indicate the power. In addition, the overall reproduction number is (*q*_*t*_ + *η*∙*p*_*t*_)∙*R*_*t*_.

For the observed sequencing data, we denote the numbers of original and mutated strains by *m*_*t*_ and *n*_*t*_, respectively, for the *t*-th day. The expected chance (or probability) that a randomly selected strain at the *t*-th day carrying a specific mutation is given in Eq. (3).

3 $$\mathbf{E}\left[{p}_{t}\right]=\frac{\eta {R}_{t}{\int }_{0}^{\infty }w\left(k\right)\cdot p\left(t-k\right)\cdot C\left(t-k\right)\mathrm{d}k}{\mathbf{E}\left[{C}_{t}\right]}.$$

Then, we have **E**[*q*_*t*_] = 1 − **E**[*p*_*t*_], which can be modelled with the same fashion. As such, by modelling the sampling of the genetic variants as a Bernoulli process, we construct the likelihood function (${L}_{t}^{(\mathrm{s})}$) of the observed genotype using a Bernoulli-distributed framework with probability at **E**[*p*_*t*_] as in Eq. (4).

4 $${L}_{t}^{(\mathrm{s})}\left({m}_{t},{n}_{t}|\mathbf{E}\left[{p}_{t}\right]\right)={(1-\mathbf{E}\left[{p}_{t}\right])}^{{m}_{t}}\cdot {\mathbf{E}\left[{p}_{t}\right]}^{{n}_{t}}.$$

Here, the superscript ‘^(s)^’ merely indicated the likelihood function is for genetic variants, which does not indicate the power.

With Eqs. (2) and (4), we reconstruct the *R*_*t*_ time series, denoted by $\left\{{R}_{t}\right\}$, and estimate *η* using the overall likelihood function defined in Eq. (5).

5 $$L\left(\left\{{R}_{t}\right\},\eta |\left\{{C}_{t}\right\},\left\{{m}_{t}\right\},\left\{{n}_{t}\right\}\right)={\prod }_{t}\left[{L}_{t}^{(\mathrm{c})}\cdot {L}_{t}^{(\mathrm{s})}\right].$$

Similar formulations were adopted in previous studies [19, 31, 32].

### COVID-19 surveillance data and SARS-CoV-2 sequencing data

**Fig. 1:**
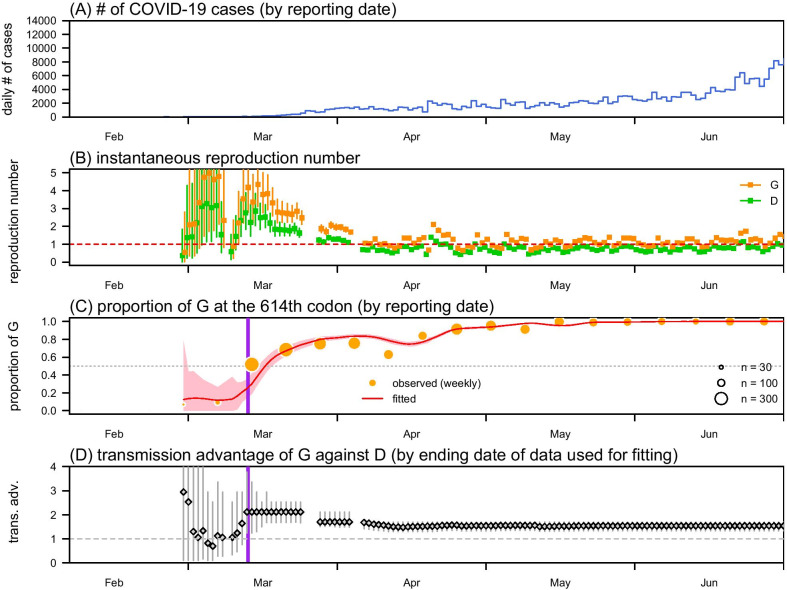
The daily number of COVID-19 cases (panel **A**), the reconstructed reproduction number (*R*_*t*_, panel **B**), proportion of the G on the 614-th codon of the S protein (panel **C**), and estimated transmission advantage of G on the real-time basis (*η*, panel **D**). Panel A shows the daily number of COVID-19 time series in California state, USA. Panel **B** shows the estimated *R*_*t*_s of G (in orange) and **D** (in green). Panel **C** shows the observed (dots) and fitted (curve) proportion of the Glycine (G) on the 614-th codon of the S protein. Panel **D** shows the real-time estimates of the transmission advantage of G (*η*). In panels **B** and **D**, the dots are the estimates, and bars are the 95%CIs. In panel **C**, the curve indicates the mean fitting results, the shading area indicates the 95%CIs, and horizontal dashed grey line represents proportion level at 0.5. In panels C and D, the vertical bold purple line represents the date, March 12, 2020 when the *η* estimates yields an ‘early warning signal’, i.e., *η* > 0 significantly, detecting the transmission advantage of G (against **D**)

To demonstrate the application of the framework, we adopted the data of COVID-19 in California, USA, and estimated the transmission advantage *η* of the D614G substitution. The surveillance data of daily number of COVID-19 cases are collected from the **R** package “*nCov2019*” [33], which is extracted from the COVID-19 surveillance platform launched by the New York Times. Figure 1A shows the daily number of COVID-19 cases time series in California.

The SARS-CoV-2 strains are obtained via the Global initiative on sharing all influenza data (GISAID) with collection dates ranging from January 1 to June 30, 2020 in California [34]. A total of 4268 full-length human SARS-CoV-2 strains are retrieved on December 31, 2020. All SARS-CoV-2 strains used for analysis are provided in the appendix (Additional file 1). We consider the study period from January 1 to June 30, 2020 when other mutated lineages, e.g., B.1.1.7, P.1, or B.1.617.2, were not yet detected. Multiple sequence alignment is performed using Clustal Omega [35], and the SARS-CoV-2 strain ‘*China/Wuhan-Hu-1/2019|EPI_ISL_402125*’ is considered as the reference sequence.

### Likelihood-based inference and real-time estimation

To setup the model, we considered the *w* as a Gamma distribution having mean (± SD) values of 5.3 (± 2.1) days by averaging the GT estimates for COVID-19 from the existing literatures [27–29, 36, 37]. Slight variation in the settings of the GT will not affect our main findings.

Using the likelihood framework defined in Eq. (5), we calculate the maximum likelihood estimation (MLE) of *η* to determine transmission advantage of D614G substitution. The 95% confidence intervals (95%CI) are calculated using the profile likelihood estimation framework with a Chi-square quantile as cutoff [38, 39], which is also adopted in [40–43].

For the real-time estimation, we repeat the statistical inference process of *η* using a part of dataset, instead of the full dataset, divided by the observing date. For example, the real-time estimate of *η* on the *τ*-th day is calculated by using the dataset with reporting date from the first day (i.e., January 1, 2020) to the *τ*-th day. We compare the consistency of the *η* estimates on a real-time basis in terms of their scales and 95%CIs. Moreover, we define the early warning signal as that a real-time estimate of *η* larger than 1 and of statistical significance can be obtained before the mutated strains (i.e., those SARS-CoV-2 strains with amino acid G) reach the dominance level in the population. For dominance level, it is considered as the proportion of the mutated strains (*p*_*t*_) over 0.5, i.e., *p*_*t*_ > 0.5, which can be observed empirically. An early warning signal indicates the real-time estimating potentials of our analytical framework in detecting the transmission advantage due to mutation.

## Results

In California, the epidemic curve grew since February, see Fig. 1A, peaked in July with daily number of COVID-19 case over 10,000, declined in August, and has maintained at a steady level since September (data not shown). We reconstruct the daily instantaneous reproduction numbers of the cases infected by SARS-CoV-2 strains with D614 or G614 type in Fig. 1B. We observe that the overall trends of reproduction numbers are relatively high in the early March, but gradually decreasing thereafter since the local ‘stay-at-home’ order was issued on March 19, 2020 in California [44]. During the first half of March, which is regarded as the early phase of the outbreak, the average reproduction number is 2.4, which is largely consistent with most of previous estimates [15, 16, 45–47].

We report the estimated proportion of D614G substitution **E**[*p*_*t*_] fits the observed sequencing data well, see Fig. 1C. We infer the transmission advantage *η* at 1.54 (95%CI: 1.36, 1.72), which means the D614G substitution increases 54% of the transmissibility. Hence, in Fig. 1B, the reproduction number of the SARS-CoV-2 variant with 614G appears higher than that of the original genotype. Although reproduction number *R*_*t*_ of the 614D are below 1 for most of the time after April 2020, the reproduction number *η*∙*R*_*t*_ of type G fluctuated around 1 during the same period, which led to a large-scale epidemic wave in California during summer in 2020 (see Fig. 1A).

For the real-time estimating potentials, we find that the real-time estimates of *η* appear unstable in February and early March, when the D614G substitution emerge, and gradually converge and stabilize since March 12, see Fig. 1D. Specially, on March 12 (highlighted in Fig. 1C and Fig. 1D), when the proportion of D614G substitution (*p*_*t*_) reaches 35% (< 0.5), the *η* estimate is 2.12 (95%CI: 1.24, 3.78), which is significantly larger than 1.

## Discussion

Although the variants carrying D614G substitution might be introduced to California from aboard during the first few months of pandemic, the observed changes in SARS-CoV-2 mutations (*p*_*t*_) were likely due to the spread of virus locally after the implementation of strict travel-ban measurements. The significant increase in transmissibility associated with the D614G substitution is biologically reasonable according to similar findings reported in previous studies. Consistent evidences of the transmission advantage of D614G substitution were also reported in previous literature both statistically [19, 48] and experimentally [49–53]. The D614G replacement leads to increased infectivity and stability of the virion and is shown to enhance viral replication in human lung epithelial cells [51, 52]. The interaction of the SARS-CoV-2 S protein with multiple epithelium components, e.g., glycocalyx, and proteases, govern the cellular entry [54]. Thus, the mutations on S protein with more effective interaction with these epithelium components enables SARS-CoV-2 variants to infect with relatively lower virus titer. Previous analysis implied that the D614G substitution may alter the conformation of spike protein trimer that shifted toward an ACE2 binding-competent state [50], and thus may functionally improve receptor binding capacity from a theoretical perspective [17, 18, 53]. The D614G substitution increases host cell entry via ACE2 and transmembrane protease serine 2 (TMPRSS2) [54]. Comparing to substitution, we learn from the influenza virus that major antigenic changes can be caused by a single AA substitution related to the receptor binding domain (RBD) [55].

Although a significant transmission advantage of D614G is found, we notice that the proportion of 614G variant generally increased, while the reproduction number series decreased in March and then remained constant. The reasons may include that the increase in transmissibility associated with D614G was counteracted by the effects of local non-pharmaceutical interventions that reduced the overall transmission of COVID-19. For sensitivity checking, we repeat the estimating process of *η* with alternative mean GT using a shorter estimate of 4.0 days [30] and a longer estimate of 7.5 days [15]. We find that the *η* estimates are consistently and significantly larger than 1 in similar scales (data not shown), which validates our main results. The statistical inference framework is empirical, and thus can be extended to explore the transmission advantage attributed to single mutations for other infectious diseases.

Our analytical framework can yield an early warning signal in detecting the transmission advantage due to D614G substitution before the mutation reaching dominance on a real-time basis. Although some recent studies indicate that the D614G mutation is unlikely to undermine the neutralization from current SARS-CoV-2 vaccine candidates [53, 56], there are also other studies suggest the concerns should be raised oppositely [57, 58]. Similar concerns of the changes in the protective effect from vaccine or prior infection are frequently raised regarding other recent SARS-CoV-2 varaints [22, 59–62]. Under selection, viral quasispecies including closely related viral genomes might be generated by the accumulation of mutations [63]. As such, the early warning signal provides an opportunity for improving disease control strategies and healthcare planning against the mutated strains, which might have different diagnostic conditions or clinical outcomes [19, 50, 53]. Hence, we highlight the importance of our analytical framework, such that the public health risks related to viral mutations may be controllable with early preparedness.

For the limitations of this study, we have the following remarks. First, the reconstruction of *R*_*t*_ relies on the setting of the generation time (GT). We model the GT distribution, i.e., *w*(∙), of COVID-19 as a fixed Gamma distribution, which follows previous studies [27–30, 36]. In the real-world situation, the time interval between transmission generations might be varying [45], which may affect the reconstruction of reproduction number. However, the overall trends of *R*_*t*_ estimates are unlikely changed due to slight variation in GT [45]. Thus, we consider the impact of this limitation on the inference of transmission advantage may be negligible, and our model can be extended to a more complex context with the time-varying GT data available. Second, theoretically, the GT distribution might be altered by the mutated strains. However, by screening the literature of COVID-19, we find no evidence that GT is varied associated with the D614G substitution in SARS-CoV-2, and thus we presume *w*(∙) to be a fixed distribution. Third, for the *R*_*t*_ estimation parts, *C*(*t*) in the ‘methods’ section should be the numbers of COVID-19 cases onset at time *t*. However, due to the surveillance data by onset date are unavailable, we adopted the current dataset by reporting data as a proxy for the COVID-19 incidence time series. If one considers a constant reporting lag, the *R*_*t*_ estimates will have exactly same trends but shifted for the reporting lag. Considering the similar reporting delay also occurred for the SARS-CoV-2 sequencing data, the effects of the two reporting lags may be counteracted. We remark that this approximation in analysis is unlikely to affect the main conclusions in this study. Furthermore, with detailed reporting lag information of each individual case, adjustment for reporting delay can surely be carried out based on our current analytical framework. Fourth, this study focuses on exploring the effects on changing the disease transmissibility associated with a single mutation, e.g., D614G, but the real-world biological mechanisms, which are usually more complex, remain uncovered. As an example, on one hand, the R384G substitution in influenza A/H3N2 virus enhances ability of in-host immune-escape [64], which indicates an increase in infectivity [9], but this substitution appears detrimental. On the other hand, the co-mutations of R384G in nucleoprotein (NP) could improve and compensate the viral fitness or functionality of [7, 8], such that the mutated strains reached fixation rapidly in 1993–1994 flu season. Future studies are needed for exploring the mechanisms of how D614G in SARS-CoV-2 affects the transmissibility of COVID-19. Fifth, the transmission advantage can be contributed by multiple factors such as increase in infectiousness or viral viability, change in the infection risk to different group of hosts [65], change in the escape from antibodies, shortening of generation interval, changes in clinical conditions, population size dynamics, and selective pressures [66]. Our analytical framework cannot disentangle the effects of each factor, which requires more complex methods, and detailed information [67]. Sixth, homogeneous mixing and equal contribution of all cases were assumed in our model. Thus, the reproduction numbers and transmission advantage estimates are interpreted as the average scales for the whole population in California. Seventh, there are multiple mutations in the SARS-CoV-2 variants carrying 614G, and we remark that the independent effects of each mutation cannot be disentangled in this study, where the interactions among these mutations are unassessed. Lastly, as a data-driven study, the estimated association should be interpreted with caution. With ecological setting, though our analysis provides statistical evidence about the likelihood of causality, the findings in this study cannot guarantee the causality, which needs further biomedical experiments in more sophisticated contexts.

## Conclusions

The modelling framework in this study links together the mutation activity at molecular scale and COVID-19 transmissibility at population scale. We report statistical evidence of the transmission advantage associated with the D614G substitution in SARS-CoV-2. We highlight that an early warning signal in detecting this transmission advantage can be generated on a real-time basis. Future studies on exploring the mechanism between SARS-CoV-2 mutation and COVID-19 infectivity are needed.

## Supplementary Information

**Additional file 1.** The acknowledgement table of SARS-CoV-2 strain sequences used in this study.

## Data Availability

All data used in this work are publicly available. The processed data and codes are available via https://github.com/plxzpnxZBD/real-time_TransAdv.

## Abbreviations

AA: Amino acid
COVID-19: Coronavirus disease 2019
D614G: The amino acid substitution changing from Aspartic Acid (D) to Glycine (G) on the 614-th codon (of the S protein of SARS-CoV-2)
GT: Generation time
LR: Likelihood-ratio
GISAID: Global initiative on sharing all influenza data
MLE: Maximum likelihood estimation
RBD: Receptor binding domain
SARS-CoV-2: Severe acute respiratory syndrome coronavirus 2
SD: Standard deviation
95%CI: 95% Confidence interval
